# Secondary Metabolite Profiling of *Satureja aintabensis* P.H. Davis and *Satureja spicigera* (K. Koch) Boiss. by LC-HRMS and Evaluation of Antioxidant and Anticholinergic Activities

**DOI:** 10.3390/life15081272

**Published:** 2025-08-11

**Authors:** Ayşe Nur Yıldız, Sema Çarıkçı, Tuncay Dirmenci, Murat Kartal, İlhami Gülcin, Ahmet C. Gören

**Affiliations:** 1 Department of Pharmacognosy, Institute of Health Sciences, Bezmialem Vakif University, Fatih, Istanbul 34093, Türkiye; eczaciaysenuryildiz@gmail.com; 2 Vocational School, Izmir Demokrasi University, Izmir 35140, Türkiye; sema.carikci@idu.edu.tr; 3 The Sustainable Environmental Studies Application and Research Centre, Izmir Demokrasi University, Izmir 35140, Türkiye; 4 Department of Biology Education, Necatibey Faculty of Education, Balikesir University, Balıkesir 10145, Türkiye; dirmenci@balikesir.edu.tr; 5 Department of Pharmacognosy, Faculty of Pharmacy, Bezmialem Vakif University, Fatih, Istanbul 34093, Türkiye; murat.kartal@bezmialem.edu.tr; 6 Phytotheraphy Research Center, Bezmialem Vakif University, Istanbul 34093, Türkiye; 7 Department of Chemistry, Faculty of Science, Atatürk University, Erzurum 25240, Türkiye; igulcin@atauni.edu.tr; 8 Rectorate of Agri Ibrahim Cecen University, Yeni Üniversite Caddesi No: 2 AE/1, Ağrı 04100, Türkiye; 9 Department of Chemistry, Faculty of Basic Sciences, Gebze Technical University, Gebze 41400, Türkiye; 10 Troyasil HPLC Column Technologies, Doruk Analitik, Mehmet Akif Mah, Yumurcak Sok, No. 43, Istanbul 34744, Türkiye

**Keywords:** *Satureja aintabensis*, *Satureja spicigera*, LC-HRMS, cholinergic activity, hesperidin, antioxidant activity, rosmarinic acid

## Abstract

In this study, phenolic compounds of methanol extracts obtained from the leaves and branches of *Satureja aintabensis* P.H. Davis and *Satureja spicigera* (K. Koch) Boiss. species were determined as mg/kg extract using the liquid chromatography high resolution mass spectrometry technique. The in vitro inhibitory effects of these extracts against enzymes associated with neurodegenerative Alzheimer’s disease (AD) were also evaluated. The relationship between secondary metabolite structures and biological activities was discussed. The major components of *S. aintabensis* were determined as hesperidin (6.465% of the extract; 64.65 g/kg), syringic acid (5.964% of the extract; 59.64 g/kg), rosmarinic acid (5.248% of the extract; 52.48 g/kg) and naringenin (0.395% of the extract; 3946.84 mg/kg), while syringic acid (3.081% of the extract; 30.81 g/kg), rosmarinic acid (2.757% of the extract; 27.57 g/kg), hesperidin (1.723% of the extract; 17.23 g/kg), and luteolin-7-*O*-rutinoside (1.682% of the extract; 16.82 g/kg) were determined in *S. spicigera*. AChE and BChE enzyme inhibition of the extracts were analyzed. The species showed moderate inhibition against AChE enzyme and low inhibition against BChE enzyme. The antioxidant properties of both plant extracts were evaluated by measuring three radical scavenging capacities and the ability to reduce Fe^3+^, and Cu^2+^ ions. *S. aintabensis* showed better antioxidant capacity in all methods except DPPH scavaging assay. These data clearly show that both species, especially *S. aintabensis*, have emerged as a new and important natural source of hesperidin, syringic acid and rosmarinic acid and an antioxidant agent for pharmaceutical and nutraceutical applications.

## 1. Introduction

Lamiaceae is represented globally by approximately 7200 species and around 240 genera [1]. The species have a wide usage area among people because of their strong aromatic properties, and they are widely used as culinary herbs (basil, mint, rosemary, sage, savory, marjoram, oregano, thyme, lavender, etc.). Considering this wide range, in Türkiye, Lamiaceae is the third largest family based on the taxon number and the fourth largest family based on the species number. The rate of endemism is 44% [2]. Many species of the genera *Origanum* L., *Satureja* L., *Salvia* L., and *Thymus* L., etc., collected from nature or cultivation areas, are used especially among the local population or others as a spice and herbal tea in Türkiye [3,4,5].

The genus *Satureja* L. is represented by 46 species in the world and in Türkiye is represented by 18 species, of which 7 are endemics. The species of the genus *Satureja* are distributed mainly in the Mediterranean basin countries, in Europe, North Africa, Morocco, Libya, Saudi Arabia, Türkiye and Caucasus [1,6]. Türkiye is one of the countries with the most species of the genus *Satureja* [2,7,8]. It has been reported that species such as *Satureja* spp., *Origanum* spp., and *Thymus* spp. are consumed as herbal tea, and these plants are also rich in essential oil content and called thyme due to their thymol/carvacrol content [9,10]. The people in the region call the *Satureja* species Sivri kekik”, Kkaya kekiği” or “Kılıç kekiği”. It is mostly used as a spice and partly as herbal tea, and its essential oil is also extracted [11,12,13]. The people in the area gather and use it to make herbal teas and spices [14]. The pharmacological properties of thirteen *Satureja* species have been investigated. The most active species among these were determined to be *S. khuzestanica*, *S. bachtiarica*, *S. montana*, and *S. hortensis*, both phytopharmacologically and clinically [13]. The aerial parts of the *Satureja* species, including flower stems and leaves, are used in the treatment of various diseases of the gastrointestinal system, to treat muscle pain, and infectious diseases, and also exhibit antispasmodic properties [15]. *Satureja* species are also commercially valuable due to these properties [16]. Among the *Satureja* species in Türkiye, *S. cuneifolia*, *S. thymbra, S. hortensis* and *S. spicigera* are collected for trade [14]. *Satureja aintabensis* P.H. Davis is an endemic species in Türkiye and known as Antep kaya kekiği [17,18]. The constituents of the essential oils of the species were investigated in two different studies [19,20], and p-cymene (33% and 59%) and thymol (32% and 17.5%) were determined as the main components in both studies. Aşkun et al. analyzed the phenolic content of methanol, ethyl acetate and petroleum ether extracts by HPLC and found rosmarinic acid and hesperidin as the main abundant components [21]. They also reported that the high content of rosmarinic acid in the extracts is the reason for the antimycobacterial activity of the plant [21]. *Satureja spicigera* (K. Koch) Boiss. is native to Iran, the North Caucasus, Transcaucasia and Türkiye. It is distributed in the Central and Eastern Black Sea regions of Türkiye and popularly called “Çorba kekiği” or “Trabzon kekiği” [17,18,19,20,21,22]. The phytochemical profile of the essential oil of the plant showed that it is rich in monoterpenes and has two chemotypes (carvacrol and thymol) [23,24,25,26,27,28,29]. In addition, these studies reported that the essential oil of the species has antimicrobial, antibacterial, antifungal, and insecticidal activity and toxicity against nine *Penicillium* species [30,31,32]. In addition to essential oil studies, the total phenolic content of the plant and antioxidant capacities according to 2,2-diphenyl-1-picrylhydrazyl (DPPH), ferric-reducing antioxidant power (FRAP) methods, and *β*-carotene-linoleic acid assays are available [33,34,35,36,37]. Gohari et al. isolated nine compounds from ethyl acetate and methanol extracts of *S. spicigera* and investigated the toxicity of these compounds on some cancer lines. Among the compounds, only 5,4′-dihydroxy-3′-methoxyflavanone-7-(6″-O-α-L-rhamnopyranosyl)-*β*-_D_-glucopyranoside, which has a flavanone structure, showed an inhibition effect against T47D (breast, ductal carcinoma) [37].

Oxygen is essential for meeting the energy demands of biological tissues. However, oxygen consumption leads to the formation of reactive oxygen species (ROS), which exert harmful effects on cells. Antioxidants protect cells from damage by neutralizing harmful free radicals, thus potentially lowering the risk of many chronic illnesses [38,39,40]. Oxidative stress refers to a condition in which the balance between ROS production and antioxidant defenses is significantly disturbed. Due to its high oxygen demand and abundance of peroxidizable substrates, the brain is particularly vulnerable to the effects of ROS. Elevated oxidative stress can cause neuronal damage, culminating in cell death, and contributes to the development of neurodegenerative diseases such as Alzheimer’s disease (AS) and Parkinson’s disease (PD)[41,42,43,44]. Alzheimer’s disease (AD) is a neurodegenerative disorder characterized by cognitive impairments such as memory loss and reasoning difficulties, resulting from various mechanisms affecting cholinergic neurotransmission in the central nervous system. Due to the increasing elderly population worldwide, it has become one of the most prevalent public health challenges [45]. The multifactorial etiology of AD has hindered complete success in clinical treatment trials targeting different pathological mechanisms. The most significant neurochemical alteration observed in AD patients is the reduction in cortical acetylcholine (ACh) levels. Since acetylcholinesterase (AChE), the enzyme responsible for ACh’s metabolic hydrolysis, is abundantly present in cholinergic synapses of both central and peripheral nervous systems, modulating ACh levels remains a key therapeutic strategy. Both natural and synthetic cholinesterase (ChE) enzyme inhibitors such as donepezil and galantamine have been reported to improve cognitive and behavioral symptoms by preventing ACh degradation [45,46,47]. Research on traditional medicinal plants rich in economically valuable bioactive compounds has surged recently [45,46,47,48,49,50,51,52]. These plants have been identified as a potential source of natural medicines, with the potential to treat a range of diseases including neurodegenerative diseases, cardiovascular diseases, cancer and diabetes. In addition, they have the capacity to significantly reduce the risk of disease. Crucially, they exhibit lower toxicity than synthetic drugs, and natural products derived from diverse plant sources generally offer superior health benefits compared to synthetic alternatives.

Polyphenols, which are found primarily in fruits, vegetables, and plants, have been associated with many biological processes, including antioxidant, anticancer, antiviral, antibacterial, anti-inflammatory, neuroprotective, and cardioprotective properties. These radicals then halt or prevent the oxidation of various biomolecules within the cell. Due to these unique properties of phenolic compounds, finding new sources of antioxidants has become important. To this end, the biological activities of methanol extracts prepared from the aerial parts of both *Satureja* species (*S. aintabensis* and *S. spicigera*), which are commonly used as spices and teas, was evaluated. Antioxidant capacity was determined by three different radical scavenging methods including (1,1-diphenyl-2-picryl-hydrazyl (DPPH), N,N-dimethyl-p-phenylenediamine radicals (DMPD^+^), and 2,2-azino-bis-3-ethylbenzthiazoline-6-sulfonic acid (ABTS^+^)) and Fe^3+^ reducing ability (FRAP) and Cu^2+^ reducing ability (CUPRAC) methods. Potential anti-Alzheimer’s disease capacity was evaluated by AChE and BChE enzyme inhibition assays. To determine the components responsible for the biological activity, the phenolic content of the extract was determined using the LC/HRMS method.

## 2. Materials and Methods

### 2.1. Chemicals

The origin and purity of the chemicals and their brands used in the study and the reference materials utilized in the investigation are as follows: Acacetin (>97% TRC Canada, Winnipeg, MB, Canada), Apigenin (>97% TRC Canada, Winnipeg, MB, Canada), Apigenin 7-glucoside (>97% EDQM CS, Strasbourg, France), Ascorbic acid (≥99% Sigma-Aldrich, St. Louis, MO, USA), Caffeic acid (≥98% Sigma-Aldrich, St. Louis, MO, USA), Caffeic Asit Phenethyl Ester (≥97% European pharmacopoeia reference standard), Chlorogenic acid Chrysin (≥96% Sigma-Aldrich, St. Louis, MO, USA), Dihydrocapsaicin (≥97% Sigma-Aldrich, St. Louis, MO, USA), Dihydrokaempferol (>97% Phytolab, Vestenbergsgreuth, Germany), Fumaric acid (≥99% Sigma-Aldrich, St. Louis, MO, USA), Hesperidin (≥98% J&K, Ladakh, India), Hispidulin (>97% TRC Canada, Winnipeg, MB, Canada), Hyperoside (>97% TRC Canada, Winnipeg, MB, Canada), Isosakuranetin (>97% Phytolab, Vestenbergsgreuth, Germany), Luteolin (95% Sigma-Aldrich, St. Louis, MO, USA), Luteolin 7-glucoside (>97% TRC Canada, Winnipeg, MB, Canada), Luteolin-7-rutinoside (>97% Carbosynth limited, Staad, Switzerland), Naringenin (≥95% Sigma-Aldrich, St. Louis, MO, USA), Naringin (≥90% Sigma-Aldrich, St. Louis, MO, USA), Nepetin (98% Supelco, Bellefonte, PA, USA), Orientin (>97% TRC Canada, Winnipeg, MB, Canada), Penduletin (>97% Phytolab, Vestenbergsgreuth, Germany), Quercetin (≥95% Sigma-Aldrich, St. Louis, MO, USA), Quercitrin (>97% TRC Canada, Winnipeg, MB, Canada), Rosmarinic acid (≥96% Sigma-Aldrich, St. Louis, MO, USA), Salicylic acid (≥98% Sigma-Aldrich, St. Louis, MO, USA), Syringic acid (≥95% Sigma-Aldrich, St. Louis, MO, USA), Vanilic acid (≥97% Sigma-Aldrich, St. Louis, MO, USA), Verbascoside (86.31% HWI ANALYTIK GMBH, Rülzheim, Germany), (−)-Epicatechin (≥90% Sigma-Aldrich, St. Louis, MO, USA), (−)-Epicatechin gallate (>97% TRC Canada, Winnipeg, MB, Canada), and (+)-trans Taxifolin (>97% TRC Canada, Winnipeg, MB, Canada).

### 2.2. Plant Material

*Satureja aintabensis* P.H. Davis was collected from Gaziantep Samköy, 37°08′07.81″ N, 37°18′56.67″ E, at an altitude of 1000 m, and *S. spicigera* (K. Koch) was collected from Şavşat-Artvin 2–3 km, 41°11′00.83″ N, 41°50′50.05″ E, at an altitude of 300 m in September 2018 by Prof. Tuncay Dirmenci (Balıkesir University, Balıkesir province, Türkiye). The herbarium sample of this species was recorded and stored in Balıkesir University Necatibey Education Faculty Herbarium with the codes TD 5210 and TD 5185, respectively.

### 2.3. Preparation of Plant Extracts

Ten grams of the plant materials was weighed and put in a 250 mL capped Schott flask once the aerial parts of *S. aintabensis* and *S. spicigera* had been allowed to dry in the shade and ground up. Then, 100 mL of solvent was added and macerated for 4 days. Every day the bottle was vented, and the mixture was filtered at the end of the 4th day. The solvent was evaporated with the help of a rotary evaporator until dryness, and crude extracts were obtained. The plants yielded 0.96 and 1.01 g of extract, respectively.

### 2.4. LC-HRMS Analysis

The phenolic compounds in the methanol extract prepared from the plants were determined by liquid chromatography–high-resolution mass spectrometry (LC-HRMS) using an Orbitrap Q-Exactive mass spectrometer (Thermo Fisher Scientific Inc., Waltham, MA, USA) coupled with a Troyasil (Istanbul, Türkiye) C18 (150 × 3 mm, particle size: 5 μm) column, following the analysis method detailed in our previous studies [42,49,51]. The validation parameters of the method used were specificity, accuracy, precision, LOD and LOQ. The results were evaluated using EURACHEM/CITAC guidelines and our previous studies. More information on uncertainty assessment procedures can be found in the previous literature [44,51,53,54,55,56,57,58,59,60].

The specificity, linearity, precision, LOD and LOQ of the LC-HRMS method and the uncertainty value of the measurement results are described in Table S1 in the supporting information [15,39,44,51,53,54,55,56,57,58,59,60,61].

### 2.5. Antioxidant Activities

#### 2.5.1. DPPH Scavenging Assay

To estimate the 2,2-diphenyl-1-picrylhydrazyl (DPPH) free radical scavenging capacity of *Satureja* extracts, the method described by Blois [62], with a minor modification [56], was used. This method is based on monitoring the bleaching of the violet color of a stable free DPPH radical at a specific wavelength (517 nm) in the presence of hydrogen atoms or antioxidant substances with electron donating properties in the sample.

#### 2.5.2. ABTS^+^ Scavenging Activity

The 2,2-azino-bis-3-ethylbenzthiazoline-6-sulfonic acid (ABTS^+^) scavenging assay was employed as a second radical scavenging technique to evaluate the extracts’ capacity to scavenge ABTS radicals. This method is based on the spectrophotometric detection of the decolorization capacity of the ABTS radical, just like DPPH. After the formation of the ABTS radical cation, plant extracts at different concentrations (10–30 μg/mL) were added to the ABTS^+^ solution. After half an hour, the absorbance of samples was measured at 734 nm against the blank for all samples. Finally, the percentage of ABTS radical scavaging was calculated. The decrease in absorbance value indicates the ABTS radical scavenging capacity of the samples [44,46,53,56].

#### 2.5.3. DMPD^+^ Scavenging Activity

The third method used to determine the antioxidant capacity of the extracts, N,N-dimethyl-p-phenylenediamine (DMPD^+^) radical scavenging effect, was performed according to the method described by Gulcin [63].

In all radical scavenging assays, the chemicals butylated hydroxyanisole (BHA), butylated hydroxytoluene (BHT), Trolox and α-tocopherol were used as standards. The antioxidant potential of the samples was evaluated by calculating IC_50_ (μg/mL) values for radical scavenging capacity and comparing them with the standards.

#### 2.5.4. Cupric Ion (Cu^2+^) Reducing Ability Assay (CUPRAC)

The ability to convert cupric ions (Cu^2+^) to cuprous ions (Cu^+^) to assess antioxidant capacity is called CUPRAC, a colorimetric technique. This process produces a colored 2,9-dimethyl-1,10-phenanthroline (Neocuprine) complex with maximum absorbance. The total antioxidant capacity is calculated by measuring the absorbance values of the color of the resulting Cu^+^-Neocuprine chelate against a reference solution at 450 nm, the characteristic wavelength [39,63].

#### 2.5.5. Ferric Ion (Fe^3+^) Reducing Ability Assay

The Fe^3+^ reducing capacity of both plant extracts was evaluated based on its ability to reduce ferric ions in the presence of potassium ferricyanide, following the previous procedure [64]. In this assay, 0.75 mL of both plant extracts at varying concentrations (15–60 µg/mL) was mixed with equal volumes of 0.2 M phosphate buffer (pH 6.6) and 1% (*w*/*w*) potassium ferrocyanide. The mixture was incubated at 50 °C for 30 min. Following incubation, 1.25 mL of 10% (*w*/*w*) trichloroacetic acid and 0.25 mL of 0.1% ferric chloride solution were added. After thorough vertexing, the absorbance of each solution was recorded at 700 nm. A blank sample, in which phosphate buffer was used instead of both plant extracts, served as the negative control [64].

### 2.6. Anticholinergic Assays

Acetylcholinesterase (AChE) and butyrylcholinesterase (BChE) enzyme inhibitions were used for anticholinergic studies. As detailed in our previous study [46], the AChE/BChE inhibitory effects of the extracts were determined in accordance with the Ellman method [65].

### 2.7. Statistical Analyses

Triplicate analyses were averaged for the experiment. Data are presented as mean ± standard deviation. Variance ANOVA including one-way analysis was realized by using Microsoft Excel (see Supplementary Materials). Using Duncan’s multiple range tests, significant differences between means were noted. *p* < 0.05 was regarded as significant, and *p* < 0.01 was very significant.

## 3. Results

### 3.1. LC-HRMS Analysis Results

Using thirty-two phenolics as standard compounds, the LC-HRMS method was utilized to identify the major phenolic components in extracts. The elucidation of phenolic compounds was carried out by comparing MS information with references, and twenty-eight compounds were determined in the methanol extract of *S. aintabensis* and thirty compounds in the methanol extract of *S. spicigera* (Table 1). Table 1 shows the number of phenolic compounds (% of the extract) determined by LC/HRMS in methanol extracts of *S. aintabensis* and *S. spicigera*. Validation parameters and LC/MS-MS method developed for the secondary metabolites of the species can be found in the Supplementary Materials.

**Figure 1:**
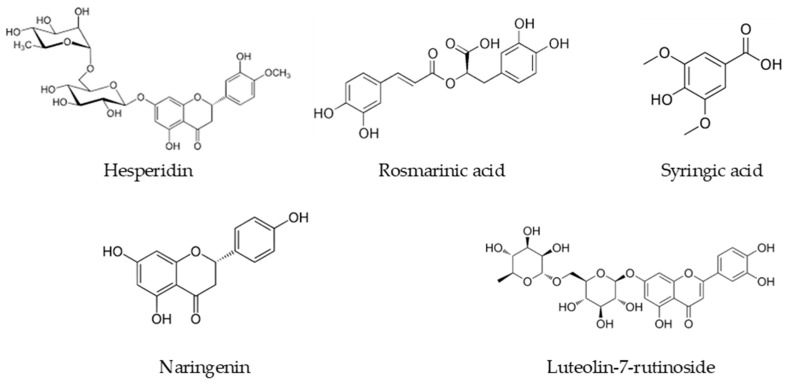
The structures of the most abundant phenolic compounds in methanol extracts of *S. aintabensis* and *S. spicigera*.

The main compounds detected in the methanol extract of *S. aintabensis* were hesperidin (6.465% of the extract; 64.65 g/kg), syringic acid (5.964% of the extract; 59.64 g/kg), rosmarinic acid (5.248% of the extract; 52.48 g/kg), naringenin (0.395% of the extract; 3946.84 mg/kg), and fumaric acid (0.244% of the extract; 2436.70 mg/kg), while (−)-epicatechin, (−)-epicatechin gallate, verbascoside and caffeic acid phenethyl ester were not determined. The main constituents of *S. spicigera* are very similar to those of *S. aintabensis*. Unlike *S. aintabensis*, syringic acid was the most abundant compound (3.081% of the extract; 30.81 g/kg), rosmarinic acid (2.757% of the extract; 27.57 g/kg) was the second, and hesperidin (1.723% of the extract; 17.23 g/kg) was the third compound. Luteolin-7-rutinoside (1.682% of the extract; 16.82 g/kg), naringin (0.674% of the extract; 6.74 g/kg), and fumaric acid (0.661% of the extract; 6.61 g/kg) were the other components with higher amounts in *S. spicigera*. Also, similar to *S. aintabensis*, (−)-epicatechin and caffeic acid phenethyl ester were not detected in the *S. spicigera* extract. The chemical structures of the most plentiful phenolics in extracts are presented in Figure 1. To create the chemical structures in Figure 1, the ChemDraw Ultra 7.0 program was utilized.

### 3.2. Reducing Ability Results

When the reduction abilities of Cu^2+^ and Fe^3+^ were examined to evaluate the antioxidant capacity, it was observed that *S. aintabensis* had better values. The reduction abilities of the standards and extracts are listed as follows.

Fe^3+^ reduction abilities: BHA (λ_593_: 2.347, r^2^: 0.9086) > Trolox (λ_593_: 2.119, r^2^: 0.9586) > α-Tocopherol (λ_593_: 0.957, r^2^: 0.9863) ≈ BHT (λ_700_: 0.952, r^2^: 0.9154) > *S. aintabensis* (λ_593_: 0.597, r^2^: 0.9618) > *S. spicigera* (λ_593_: 0.421, r^2^: 0.9236) (Table 2). Cu^2+^ reduction abilities: BHA (λ_450_: 1.649, r^2^: 0.9584) > Trolox (λ_450_: 1.108, r^2^: 0.9910) > *S. aintabensis* (λ_450_: 1.016, r^2^: 0.9954) > BHT (λ_450_: 0.998, r^2^: 0.9834) > *S. spicigera* (λ_450_: 1.016, r^2^: 0.9954) > α-Tocopherol (λ_450_: 0.693, r^2^: 0.9934).

### 3.3. Radical Scavenging Ability Results

The radical scavenging activity results of methanol extracts of the plants and positive antioxidants such as BHA, BHT, α-Tocopherol and Trolox are summarized as IC_50_ values in Table 3.

As demonstrated in Table 3, a comparison of the radical scavenging activity of the extracts reveals that the values obtained for the plants are comparable to those of BHA and α-tocopherol, as determined by the DPPH and ABTS methods. But this cannot be said for the DMPD method. In this method, the IC_50_ value for BHA, BHT and α-Trolox was approximately 0.070, while it was determined as 30.13 and 33.00 for *S. aintabensis* and *S. spicigera*, respectively.

Both extracts and standard antioxidants’ DPPH· scavenging IC_50_ values decreased in the order listed below: Trolox (IC_50_: 7.05 μg/mL, r^2^: 0.9614) > BHA (IC_50_: 10.10 μg/mL, r^2^: 0.9015) > α-Tocopherol (IC_50_: 11.31 μg/mL, r^2^: 0.9642) > *S. spicigera* (IC_50_: 12.37 μg/mL, r^2^: 0.9996) > *S. aintabensis* (IC_50_: 13.07 μg/mL, r^2^: 0.9426) > BHT (IC_50_: 25.95 μg/mL, r^2^: 0.9221).

The following range was found for the IC_50_ values of ABTS^·+^ scavenging of extracts and references (Trolox, α-Tocopherol, BHT, and BHA): BHA (IC_50_: 5.07 μg/mL, r^2^: 0.9356) > Trolox (IC_50_: 6.16 μg/mL, r^2^: 0.9692) > BHT (IC_50_: 6.99 μg/mL, r^2^: 0.9350) > α-Tocopherol (IC_50_: 8.37 μg/mL, r^2^: 0.9015) > *S. aintabensis* (IC_50_: 8.77 μg/mL, r^2^: 0.9478) > *S. spicigera* (IC_50_: 9.49 μg/mL, r^2^: 0.9343).

### 3.4. AChE and BChE Inhibition Results

In Table 4, the inhibition percentages of the AChE and BChE enzymes (which are related enzymes to AD) of methanol extracts are given. Galantamine was used as the standard.

The AChE enzyme (31.8 ± 2.2% for *S. aintabensis* and 39.7 ± 1.6% for *S. spicigera*) was inhibited relatively weaker by both plant extracts (at the 40 µg/mL extract) when compared to the BChE enzyme (9.4 ± 1.4% for *S. aintabensis* and 1.3 ± 0.7% for *S. spicigera*). On the other hand, galantamine inhibited both cholinergic enzymes at the same concentration as 96.8 ± 1.3% and 83.3 ± 0.7%, respectively.

## 4. Discussion

The antioxidant capacity of plants is determined by phenolic compounds, which can be divided into four major types based on the amount of phenol rings they contain and the structures that link them: phenolic acids, flavonoids, stilbenes, and lignans. It is known that plants, especially medicinal plants, contain over eight thousand different types of phenolic chemicals because of this structural variety. Many biological processes, including antioxidant, anticancer, antiviral, antibacterial, anti-inflammatory, and neuroprotective and cardioprotective properties, have been linked to polyphenols, which are mostly found in fruits and vegetables or plants. Because they include phenolic and polyphenolic groups, they can take up an electron to create comparatively stable phenoxy radicals, which stop or inhibit the oxidation of different biomolecules within the cell. Because of these unique properties of phenolic substances, it is important to find new sources of antioxidants [50,52]. For this purpose, two *Satureja* species commonly used as spices and teas in Türkiye were analyzed by LC-HRMS. This study revealed the antioxidant capacity of methanol extracts prepared from plants by five different methods, including radical scavenging and metal reduction principle, and determined their anti-AD effects. Phenolic compounds, which are the most important secondary metabolite group in the structure of the plant and show activity effect, were also determined. The results of the study showed that the *S. aintabensis* plant has important potential in terms of antioxidant capacity thanks to its rich phenolic content. When the literature was examined, no study was found in which the phenolic content of plants has been examined as broadly as the standards used in this study, and in narrower studies, it is possible to find some structures that have not been identified so far in the extracts of *S. aintabensis*. For *S. spicigera*, this is the first study involving the analysis of phenolic compounds.

Phenolic compounds, which are among the principal classes of secondary metabolites in plants, are commonly found in foods and are known for their antioxidant properties. Even at low concentrations, they can effectively prevent oxidative rancidity in food products. The antioxidant efficiency of phenolic compounds is largely influenced by the number and positioning of hydroxyl groups on their aromatic rings. These compounds generally exhibit antioxidant activity when hydroxyl groups are located at the *ortho*- or *para*- positions, which enhances electron density and lowers the bond dissociation energy of the O–H bond, thereby improving reactivity against lipid-derived free radicals. In contrast, substitution at the meta-position has a minimal impact on antioxidant performance. Both steric hindrance and electronic factors contribute to the antioxidant capacity and effectiveness of phenolics as chain-breaking agents. Molecular orbital theory has been utilized to understand the mechanism by which phenolic antioxidants donate hydrogen atoms during the lipid autoxidation process. More recently, brominated phenols have gained attention for their diverse pharmacological effects, including notable antioxidant activity [53].

According to the results of the LC-HRMS analysis, the phenolic substances determined in the extracts were grouped as flavonoids and derivatives, coumaric acid and derivatives, simple phenolic and other organic substances according to their chemical structures. In both plant species, flavonoids and their derivatives dominated in number and amount (78.628 g/kg for *S. aintabensis*, 44.337 g/kg for *S. spicigera*). While hesperidin, a flavanone glycoside, was determined as the most abundant component for *S. aintabensis*, syringic acid, a benzoic acid derivative, was determined as the main component for *S. spicigera*. When the studies in the literature were examined, it was reported that hesperidin is a molecule with antioxidant and anti-inflammatory activity [66,67]. Recently, 31.9 g/kg, 29.6 g/kg and 56.3 g/kg total secondary metabolites were detected in dichloromethane, acetone and methanol extracts of *S. pilosa* leaf, respectively [39]. An interesting feature of hesperidin, one of the most important citrus flavonoids, is its therapeutic effect on vascular diseases such as easy bruising and varicose veins. Anti-hypertensive and diuretic effects have also been identified. Among the plant species studied, *S. aintabensis* contains high levels of hesperidin and when the antioxidant activity was examined, it was observed that it showed greater activity than *S. spicigeria*.

In a recent study, the IC_50_ value of DPPH free radical scavenging for methanol extract of *Satureja icarica* was calculated as 10.19 μg/mL. The same value was found for 5.07 μg/mL against ABTS radicals and 26.65 μg/mL towards DMPD radicals. Similarly, methanol extract of *S. icarica* exhibited absorbance values of 0.703 and 1.056 for Fe^3+^ and Cu^2+^ ion reducing abilities [15]. The dichloromethane extract of *S. pilosa* leaves exhibited Cu^2+^ ion reducing capacity with a value of 1.13 ± 0.04 mmol Trolox/g. In contrast, the methanol extract of the leaves demonstrated a significantly higher reducing capacity, reaching 2.40 ± 0.07 mmol Trolox/g. Notably, the dichloromethane extract of the branches showed nearly a 50% reduction in activity compared to the leaf extract. Among the branch extracts, the acetone extract displayed the highest Cu^2+^ ion reducing capacity [39].

Oxidative stress has been demonstrated to play a crucial role in the formation of lesions caused by toxic substances, which are known to promote the development of Alzheimer’s disease (AD). Because of phenolic substances with antioxidant properties, the reduction in oxidative stress may lead to therapeutic effects that affect apoptosis and cell viability in cells, preventing neurodegenerative diseases such as AD. The pathogenicity of AD is multifactorial, involving vascular dysfunction, mitochondrial dysfunction due to the overproduction of reactive oxygen species (ROS), and a combination of genetic and environmental factors. Some studies have tried to explain the mechanism by which oxidative stress contributes to the development of AD. In one study, it was shown that oxidative stress causes the conversion of soluble amyloid into insoluble fibril form, which contributes to the progression of AD, and in another study, it was shown that the oxidation of Tau proteins, one of the characteristic features of AD, by free radicals in vitro may cause dimerization and polymerization of this protein [68,69,70,71,72,73]. Preventing the decrease in cortical acetylcholine levels observed in AD patients is one of the strategies in the treatment of the disease, and therefore the inhibition of cholinesterase enzymes (AChE/BChE), the enzyme responsible for the metabolic hydrolysis of acetylcholine, remains an important therapeutic strategy [45,46,47]. Antioxidants are widely preferred to combat oxidative stress. These substances are typically absorbed into the body through the incorporation of natural sources into the diet. Recent studies have demonstrated an increased efficacy of diets containing a combination of antioxidants in conjunction with a nutrient rich in antioxidants in combating the pathogenesis of AD, as they may play an important role in delaying the onset of AD as well as reducing its progression. The inhibition effect of *S. aintabensis* species with higher antioxidant capacity against AChE enzyme also supports this theory.

Acetylcholinesterase (AChE) and butyrylcholinesterase (BChE) are enzymes responsible for the hydrolysis of choline-based esters. AChE is primarily found in nerve synapses and plays a critical role in terminating synaptic transmission by breaking down the neurotransmitter acetylcholine. BChE, while similar in function, is mainly found in the liver and plasma and has broader substrate specificity. Both enzymes are targets in the treatment of neurodegenerative diseases such as Alzheimer’s disease, where inhibition can help increase acetylcholine levels in the brain. Moreover, their activity is important in toxicology, particularly in organophosphate poisoning [74]. Recently, many studies have been conducted to investigate the inhibitory effects of Satureja species on both cholinergic enzymes. In these, water and methanol extracts of *Satureja cuneifolia* demonstrated an effective inhibition effect on both enzymes, with IC_50_ values in the range of 23.17± 93.58 µg/mL [40]. According to a recent study, the strongest AChE inhibitory effect was observed in the methanol extract of the leaves of *S. pilosa*, with an IC_50_ value of 41.2 ± 5.60 μg/mL. In contrast, the highest BChE inhibition was found in the dichloromethane extract of the leaves of *S. pilosa*, showing IC_50_ values within 52.3 ± 8.6 μg/mL. For comparison, galantamine exhibited IC_50_ values of 4.1 ± 0.2 μg/mL for AChE and 12.3 ± 0.3 μg/mL for BChE. These findings suggest that the anticholinergic activity of the species is primarily attributed to rosmarinic acid and other catechol-type phenolic constituents [39].

In conclusion, in this study, both *Satureja* species were identified as important antioxidant sources with their rich phenolic contents, and the cholinergic activities study, which was conducted for the first time for these species, contributed to the explanation of the relationship between phenolic compounds with high antioxidant capacity and AD.

## Figures and Tables

**Table 1:** The quantity of phenolic compounds determined in methanol extracts of *S. aintabensis* and *S. spicigera* (% g/1000 g of extract) by LC/HRMS.

Compounds	*Satureja aintabensis*	*Satureja spicigera*	U% (k = 2) *
**Flavonoids and Derivatives**			
Apigenin	0.040	0.006	11.54
Chrysin	<LOD **	<LOD **	11.09
Luteolin	0.110	0.026	12.41
Luteolin-7-rutinoside	0.065	1.682	11.45
Luteolin-7-glucoside	0.009	0.126	11.29
Apigenin-7-glucoside	0.003	0.004	11.9
Orientin	0.015	0.007	11.47
Acacetin	0.108	0.018	11.36
Hispidulin	0.048	0.005	11.23
Nepetin	0.014	<LOD **	11.24
Penduletin	0.006	0.002	11.81
Quercetin	0.026	0.005	11.42
Hyperoside	0.163	0.044	11.5
Quercitrin	0.010	0.010	11.69
(−)-Epicatechin	<LOD **	<LOD **	11.91
(−)-Epicatechin gallate	<LOD **	<LOD **	11.21
(+)-*trans* Taxifolin	0.162	0.031	11.19
Dihydrokaempferol	0.125	0.032	11.35
Naringenin	0.395	0.034	11.04
Isosakuranetin	0.021	0.001	11.48
Naringin	0.077	0.674	12
Hesperidin	**6.465**	**1.723**	11.15
**Coumaric acids and Derivatives**			
Caffeic acid	0.028	0.037	11.07
Chlorogenic acid	0.110	0.020	11.14
Rosmarinic acid	**5.248**	**2.757**	11.63
Caffeic acid phenethyl ester	<LOD **	<LOD **	11.38
**Simple Phenolics and Others** ***			
Syringic acid	**5.964**	**3.081**	12.37
Salicylic acid	0.022	0.034	11.4
Vanilic acid	0.157	0.172	11.61
Verbascoside	<LOD **	0.045	12.08
Ascorbic acid	0.064	0.047	11.07
Fumaric acid	0.243	0.661	11.14

* U% (k = 2): Uncertainty calculated using a coverage factor of k = 2. ** LOD: Limit of detection; *** single ring phenolics, organic acids, non-flavonoids.

**Table 2:** Fe^3+^ and Cu^2+^ reduction abilities of extracts and standards at 30 μg/mL concentration.

Antioxidants	Fe^3+^ Reducing		Cu^2+^ Reducing	
	λ (593 nm)	r^2^	λ (450 nm)	r^2^
BHA	2.347	0.9086	1.649	0.9584
BHT	0.952	0.9154	0.998	0.9834
Trolox	2.119	0.9586	1.108	0.9910
α-Tocopherol	0.957	0.9863	0.693	0.9934
*S. aintabensis*	0.597	0.9618	1.016	0.9954
*S. spicigera*	0.421	0.9236	0.757	0.9999

**Table 3:** IC_50_ (μg/mL) values for DPPH·, ABTS^+^ and DMPD^+^ scavenging activities of extracts and standard antioxidants.

Antioxidants	DPPH·Scavenging *		ABTS^+^ Scavenging *		DMPD^+^ Scavenging *	
	IC_50_	r^2^	IC_50_	r^2^	IC_50_	r^2^
BHA	10.10	0.9015	5.07	0.9356	0.070	0.9465
BHT	25.95	0.9221	6.99	0.9350	0.070	0.9390
Trolox	7.05	0.9614	6.16	0.9692	0.072	0.9382
α-Tocopherol	11.31	0.9642	8.37	0.9015	-	-
*S. aintabensis*	13.07	0.9426	8.77	0.9478	30.13	0.9804
*S. spicigera*	12.37	0.9996	9.49	0.9343	33.00	0.9254

‎* *p* < 0.05 was regarded as significant, *p* < 0.01 was very significant.‎

**Table 4:** Acetylcholinesterase (AChE), and butyrylcholinesterase (BChE) enzymes’ inhibition (%) of the extracts (40 µg/mL).

Inhibitors	AChE Inhibition (%) *	BChE Inhibition (%) *
*Satureja aintabensis*	31.8 ± 2.2	9.4 ± 1.4
*Satureja spicigera*	39.7 ± 1.6	1.3 ± 0.7
Galantamine	96.8 ± 1.3	83.3 ± 0.7

‎* *p* < 0.05 was regarded as significant, *p* < 0.01 was very significant.‎

## Data Availability

All data related to the study are provided in the article.

## Supplementary Materials

The following supporting information can be downloaded at https://www.mdpi.com/article/10.3390/life15081272/s1, S1. Chemical impurity, S2. LC-HRMS analysis, Table S1: Validation parameters and LC/MS-MS method developed for the secondary metabolites of the species, File S1: ANOVA: Single Factor, Figure S1: LC-HRMS chromatogram of *Satureja aintabensis* (MeOH) extract, Figure S2: LC-HRMS chromatogram of *Satureja spicigera* (MeOH) extract, Figure S3: LC-HRMS chromatogram of internal standard (dihydrocapsaicin).

## Abbreviations

The following abbreviations are used in this manuscript:

AD	Alzheimer’s disease
AChE	Acetylcholinesterase enzyme
BChE	Butyrylcholinesterase enzyme
BHA	Butylated hydroxyanisole
BHT	Butylated hydroxytoluene
DPPH	2,2-diphenyl-1-picrylhydrazyl
ABTS	2,2-azino-bis-3-ethylbenzthiazoline-6-sulfonic acid
DMPD	N,N-dimethyl-p-phenylenediamine
CUPRAC	Cupric Ions (Cu^2+^) Reducing Ability Assay
IC_50_	Half-maximal inhibitory concentration
