# Supplementary material for: Secondary Metabolite Profiling of Satureja aintabensis P.H. Davis and Satureja spicigera (K. Koch) Boiss. by LC-HRMS and Evaluation of Antioxidant and Anticholinergic Activities
Source: Life (Basel). 2025 Aug 11;15(8):1272. doi: 10.3390/life15081272 (PMC12387306; doi:10.3390/life15081272)
Supplement: Supplementary file 1 [file life-15-01272-s001.zip › life-3745573-supplementary.pdf]

Supporting Information

Secondary Metabolite Profiling of *Satureja aintabensis* P.H. Davis and *Satureja spicigera* (K. Koch) Boiss. by LC-HRMS and Evaluation of Antioxidant and Anticholinergic Activities

Ayşe Nur Yıldız [1], Sema Çarıkçı [2,3], Tuncay Dirmenci [4], Murat Kartal [5,6], İlhami Gülcin [7,8] and Ahmet C. Gören [9,10,*]

[1] Department of Pharmacognosy, Institute of Health Sciences, Bezmialem Vakif University, Fatih, Istanbul 34093, Türkiye; eczaciaysenuryildiz@gmail.com
[2] Vocational School, Izmir Demokrasi University, Izmir 35140, Türkiye; sema.carikci@idu.edu.tr
[3] The Sustainable Environmental Studies Application and Research Centre, Izmir Demokrasi University, Izmir 35140, Türkiye
[4] Department of Biology Education, Necatibey Faculty of Education, Balikesir University, Balıkesir 10145, Türkiye; dirmenci@balikesir.edu.tr
[5] Department of Pharmacognosy, Faculty of Pharmacy, Bezmialem Vakif University, Fatih, Istanbul 34093, Türkiye; murat.kartal@bezmialem.edu.tr
[6] Phytotheraphy Research Center, Bezmialem Vakif University, Istanbul 34093, Türkiye
[7] Department of Chemistry, Faculty of Science, Atatürk University, Erzurum 25240, Türkiye; igulcin@atauni.edu.tr
[8] Rectorate of Agri Ibrahim Cecen University, Yeni Üniversite Caddesi No: 2 AE/1, Ağrı 04100, Türkiye
[9] Department of Chemistry, Faculty of Basic Sciences, Gebze Technical University, Gebze 41400, Türkiye
[10] Troyasil HPLC Column Technologies, Doruk Analitik, Mehmet Akif Mah, Yumurcak Sok, No. 43, Istanbul 34744, Türkiye
* Correspondence: ahmet.goren@acgpubs.org or acgoren@gtu.edu.tr

Table of Contents	Page
**S1.** Chemical Impurity	2
**S2.** LC-HRMS Analysis	3
**Table S1**: Validation parameters and LC/MS-MS method developed for the secondary metabolites of the species	5
**Figure S1.** LC-HRMS chromatogram of *Satureja aintabensis* (MeOH) extract	6
**Figure S2:** LC-HRMS chromatogram of *Satureja spicigera* (MeOH) extract	8
**Figure S3**. LC-HRMS chromatogram of internal standard (dihydrocapsaicin)	10

S1. *Chemical Impurity*

Ascorbic acid (≥99 % Sigma-Aldrich) , Chlorogenic acid, Fumaric acid (≥99 % Sigma-Aldrich), (-)-Epicatechin (≥90 % Sigma-Aldrich), (-)-Epicatechin gallate (>97% TRC Canada), Verbascoside (86.31% HWI ANALYTIK GMBH), Orientin (>97% TRC Canada), Caffeic acid (≥ 98 % Sigma-Aldrich , (+)-trans taxifolin (>97% TRC Canada), Luteolin-7-rutinoside (>97% Carbosynth limited) , Vanilic acid (≥97 % Sigma-Aldrich), Naringin (≥90 % Sigma-Aldrich), Luteolin 7-glucoside (>97% TRC Canada), Hesperidin (≥ 98% J&K), Rosmarinic acid (≥96 % Sigma-Aldrich), Hyperoside (>97% TRC Canada), Dihydrokaempferol (>97% Phytolab), Apigenin 7-glucoside (>97% EDQM CS), Quercitrin (>97% TRC Canada), Quercetin (≥95% Sigma-Aldrich), Salicylic acid (≥98 % Sigma-Aldrich), Naringenin (≥95 % Sigma-Aldrich), Luteolin (95% Sigma-Aldrich), Nepetin (98% Supelco), Apigenin (>97% TRC Canada), Hispidulin (>97% TRC Canada), Isosakuranetin (>97% Phytolab), Penduletin (>97% Phytolab), Caffeic Asit Phenethyl Ester (≥97% european pharmacopoeia reference standard), Chrysin (≥96% Sigma-Aldrich), Acacetin (>97% TRC Canada), Syringic acid (≥95 % Sigma-Aldrich), Dihydrocapsaicin(≥ 97 % Sigma-Aldrich)

S2. LC-HRMS Analysis

S2.1. Sample Preparation for LC-HRMS

The LC-HRMS analysis was carried out by following our previous studies [42, 49,51]. Approximately 200 mg of the plant extract was weighed and added to a 5 mL volumetric flask and 3.5 mL of methanol was added and vortexed, placed in an ultrasonic bath at 24°C and kept until a clear mixture was obtained. A 200 µL of 1000 ppm dihydrocapsaicin solution used as an internal standard was added, and the final volume was completed with methanol. After being kept in the ultrasonic bath for 10 minutes, the solutions were kept at room temperature (24 ± 3°C) in the dark for 10 minutes, filtered through a 0.45 µm Millipore Millex-HV filter and each sample was placed in 1.5 mL vials, from which 2 µL of sample was injected into the LC-HRMS device for each run.

S2.2. Standard Solutions

Standard solutions dissolved in methanol were prepared at 10 different concentrations (0.01, 0.05, 0.1, 0.3, 0.5, 1, 3, 5, 7 and 10 mg/L). A stock solution of 1000 mg/L dihydrocapsaicin (purity 97%) in methanol was used as internal standard.

S2.3. LC-HRMS Conditions

Secondary metabolites of the *Satureja* species were determined by using liquid chromatography-high-resolution mass spectrometry (LC-HRMS), which utilised an Orbitrap Q-Exactive mass spectrometer (Thermo Fisher Scientific Inc., Waltham, MA, USA) coupled with a Troyasil (Istanbul, Turkey) C18 column (150 x 3 mm, 5 µm particle size). In electrospray ionisation (ESI) mode, a combination of 1% formic acid and water (mobile phase A) and 1% formic acid and methanol (mobile phase B) was used as mobile phase. A gradient programme consisting of 90% A and 10% B for the first 60 s, 10% A and 90% B for the 7-14 min interval and 90% A and 100% B for the 14-22 min interval was used. A mobile phase with a flow rate of 0.25 mL per minute and a column with a temperature set to 25 °C were used. Environmental conditions were recorded as follows: room temperature 23.0 ± 3.0 °C and relative humidity (50 ± 15) % rh. The MS conditions used were as follows: sheath gas (Arb) 45, auxiliary gas (Arb) 10, positive ion voltage 3.50 kV, ion transfer tube temperature 300°C and evaporator temperature 320°C. Identifications were made by comparing the retention times and target ions of the compounds in LC-ESI-HRMS [42,49,51-55].

S2.4. Method Validation

The method validation parameters used in this study were specificity, accuracy, precision, LOD and LOQ. The EURACHEM/CITAC guide and our previous studies were used to evaluate sources and quantify results [42,49,51-57]. Further information on the procedures for evaluating uncertainty can be found in the previous literature [42,49,51-57].

The uncertainty value of measurement results is described in Table S1 in supporting information.

S2.4.1. Specificity

Specificity can be defined as the observation of only analyte peaks at the retention time of the target analyte in the presence of other components, such as impurities, matrix components, and degradation products. The target analyte is measured with a high degree of precision and accuracy, and is identified in the matrix without any interference. The specificity of the developed LC-ESI-HRMS method was determined by direct analysis (blind) of the entire prepared different solvents,

Satureja extracts and added target analytes. The LC-HRMS method was selected in order to achieve the required selectivity and sensitivity in the matrix and to eliminate the negative effects of the interventions.

S2.4.2. Linearity, Accuracy, LOD and LOQ of the LCHRMS Method

In its simplest form, accuracy refers to how close the measurement is to the target reference value, i.e. the difference between the observations/measurements and the actual value. The percentage recovery value for each target analyte is one of the parameters controlled to ensure accuracy. This value was calculated from LC-ESI-HRMS data for each analyte according to the following formula:

Recovery % = Recovered concentration /injected concentration x 100

The range of recovery percentages for the studied compounds was between 81.55 and 101.91 percent.

Calibration curves based on the analyte results obtained by six replicate measurements using solutions of various concentrations were used for the quantitative determination of secondary metabolites quantified by LC-ESI-HRMS. The regression coefficient (R^2) and linear regression equation obtained from the determined curve are given in Table S1.

Limits of detection (LOD), limit of quantification (LOQ) of the method for every compound were determined using the following equation: LOD or LOQ = κSDa/b, where 3 for LOQ and κ= 3 for LOD.

S2.4.3. Measurement Uncertainty Assessment

The uncertainty parameters were determined as the uncertainty due to the purity of the standard, weighing, precision and calibration curve for the applied method and the uncertainty measurement was estimated by applying the GUM methodology in accordance with EURACHEM CITAC and ISO Guide 35 [46]. Detailed equations suitable for the detailed calculation method are given in our previous studies [3, 40-45, 47-49].

Table S1: Validation parameters and LC/MS-MS method developed for the secondary metabolites of the species

Compounds	Formula	m/z	Ionization mode	Linear range	Linear regression equation	LOD / LOQ	R^2	Recovery (%)
Ascorbic acid	$C_6H_8O_6$	175.0248	Negative	0.5-10	y=0.00347x-0.00137	0.39/1.29	0.999	96.2
Chlorogenic acid	$C_{16}H_{18}O_9$	353.0878	Negative	0.05-10	y=0.00817x+0.000163	0.02/0.06	0.999	96.68
Fumaric acid	$C_4H_4O_4$	115.0037	Negative	0.1-10	y=0.00061x-0.0000329	0.05/0.17	0.999	97.13
(-)-Epicatechin	$C_{15}H_{14}O_6$	289.0718	Negative	0.05-10	y=0.0172x+0.0002269	0.01/0.03	0.999	95.66
(-)-Epicatechin gallate	$C_{22}H_{18}O_{10}$	441.0827	Negative	0.05-10	y=0.00788x-0.0001875	0.01/0.03	1.000	96.54
Verbascoside	$C_{29}H_{36}O_{15}$	623.1981	Negative	0.1-10	y=0.00758x+0.000563	0.03/0.1	1.000	96.19
Orientin	$C_{21}H_{20}O_{11}$	447.0933	Negative	0.1-10	y=0.00757x+0.000347	0.01/0.03	0.999	96.22
Caffeic acid	$C_9H_8O_4$	179.0350	Negative	0.3-10	y=0.0304x+0.00366	0.08/0.27	0.999	94.51
(+)-*trans* taxifolin	$C_{15}H_{12}O_7$	303.0510	Negative	0.3-10	y=0.0289x+0.00537	0.01/0.03	0.998	91.66
Luteolin-7-rutinoside	$C_{27}H_{30}O_{15}$	593.1512	Negative	0.1-10	y=0.00879x+0.000739	0.01/0.03	0.999	93.05
Vanilic acid	$C_8H_8O_4$	167.0350	Negative	0.3-10	y=0.00133x+0.0003456	0.1/0.33	1.000	98.66
Naringin	$C_{27}H_{32}O_{14}$	579.1719	Negative	0.05-10	y=0.00576x-0.000284	0.01/0.03	0.999	101.91
Luteolin 7-glucoside	$C_{21}H_{20}O_{11}$	447.0933	Negative	0.1-7	y=0.0162x+0.00226	0.01/0.03	0.996	96.31
Hesperidin	$C_{28}H_{34}O_{15}$	609.1825	Negative	0.05-10	y=0.00423x+0.0000138	0.01/0.03	0.999	96.14
Syringic acid	$C_9H_{10}O_5$	197.0456	Negative	0.5-10	y=0.0000831x+0.000024	0.1/0.3	0.999	97.29
Rosmarinic acid	$C_{18}H_{16}O_8$	359.0772	Negative	0.05-10	y=0.00717x-0.0003067	0.01/0.03	0.999	99.85
Hyperoside	$C_{21}H_{20}O_{12}$	463.0882	Negative	0.05-10	y=0.0072x-0.00003096	0.01/0.03	1.000	96.62
Dihydrokaempferol	$C_{15}H_{12}O_6$	287.0561	Negative	0.3-7	y=0.0756x+0.0118	0.01/0.03	0.995	95.37
Apigenin 7-glucoside	$C_{21}H_{20}O_{10}$	431.0984	Negative	0.3-7	y=0.0246x+0.00306	0.01/0.03	0.996	96.07
Quercitrin	$C_{21}H_{20}O_{11}$	447.0933	Negative	0.05-10	y=0.0179+0.0003331	0.01/0.03	0.999	97.0
Quercetin	$C_{15}H_{10}O_7$	301.0354	Negative	0.1-10	y=0.0509x+0.00467	0.01/0.03	0.998	96.41
Salicylic acid	$C_7H_6O_3$	137.0244	Negative	0.3-10	y=0.0361x+0.00245	0.01/0.03	0.998	92.88
Naringenin	$C_{15}H_{12}O_5$	271.0612	Negative	0.1-10	y=0.0281x+0.00182	0.01/0.03	1.000	86.65
Luteolin	$C_{15}H_{10}O_6$	285.0405	Negative	0.1-10	y=0.117x+0.00848	0.01/0.03	0.998	96.68
Nepetin	$C_{16}H_{12}O7$	315.0510	Negative	0.05-10	y=0.0853x+0.00269	0.01/0.03	0.999	97.76
Apigenin	$C_{15}H_{10}O_5$	269.0456	Negative	0.3-10	y=0.104x+0.0199	0.01/0.03	1.000	81.55
Hispidulin	$C_{16}H_{12}O_6$	301.0707	Pozitif	0.05-10	y=0.02614x+0.0003114	0.01/0.03	0.999	98.36
Isosakuranetin	$C_{16}H_{14}O_5$	285.0769	Negative	0.05-10	y=0.0235x+0.000561	0.01/0.03	0.999	96.56
Penduletin	$C_{18}H_{16}O_7$	343.0823	Negative	0.3-10	y=0.0258x+0.00253	0.01/0.03	0.999	83.43
Caffeic asit phenethyl ester	$C_{17}H_{16}O_4$	283.0976	Negative	0.3-7	y=0.255x+0.0477	0.01/0.03	0.996	94.42
Chrysin	$C_{15}H_{10}O_4$	253.0506	Negative	0.05-7	y=0.0964x-0.0002622	0.01/0.03	0.999	87.92
Acacetin	$C_{16}H_{12}O_5$	283.0612	Negative	0.05-7	y=0.046x+0.0001875	0.01/0.03	1.000	87.52

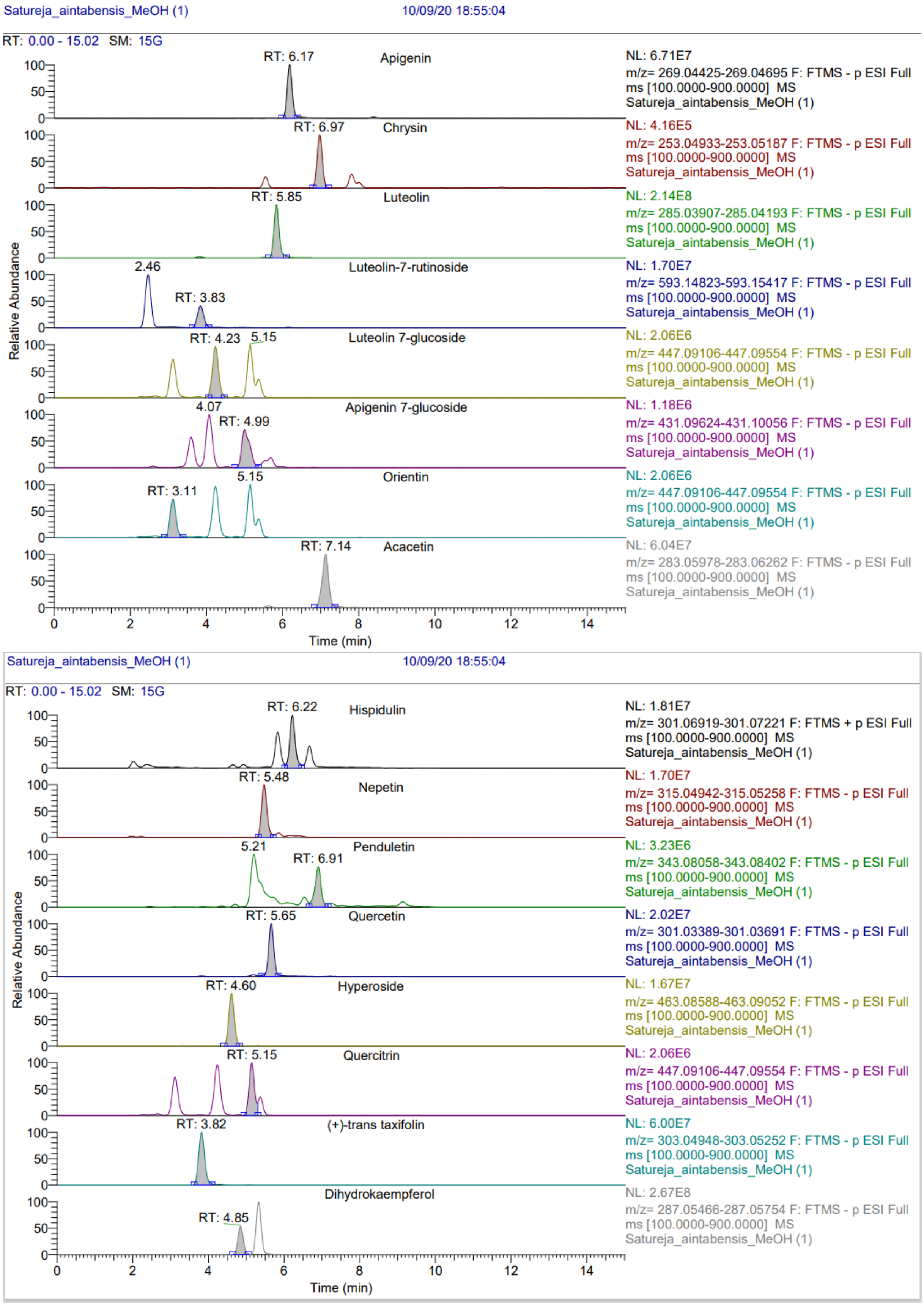

Figure S1. LC-HRMS chromatogram of *Satureja aintabensis* (MeOH) extract

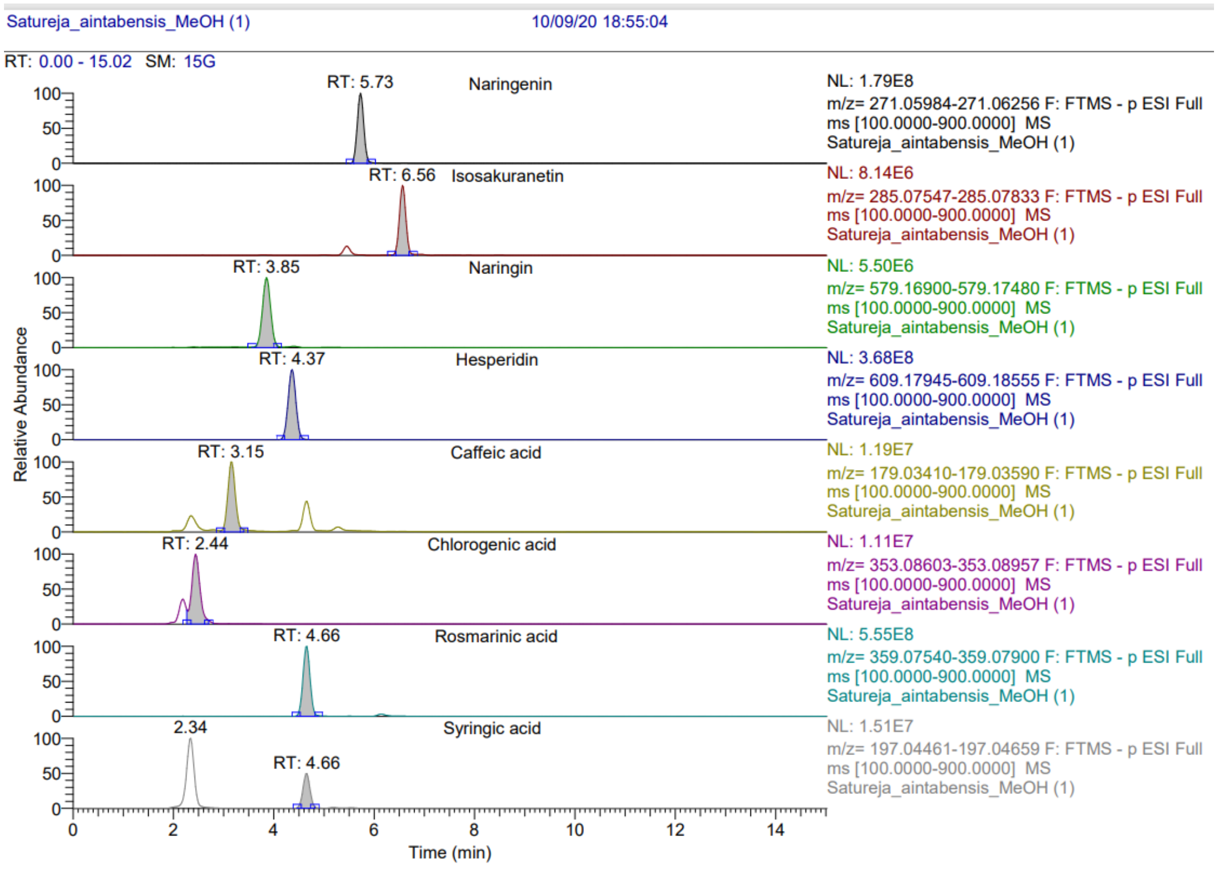

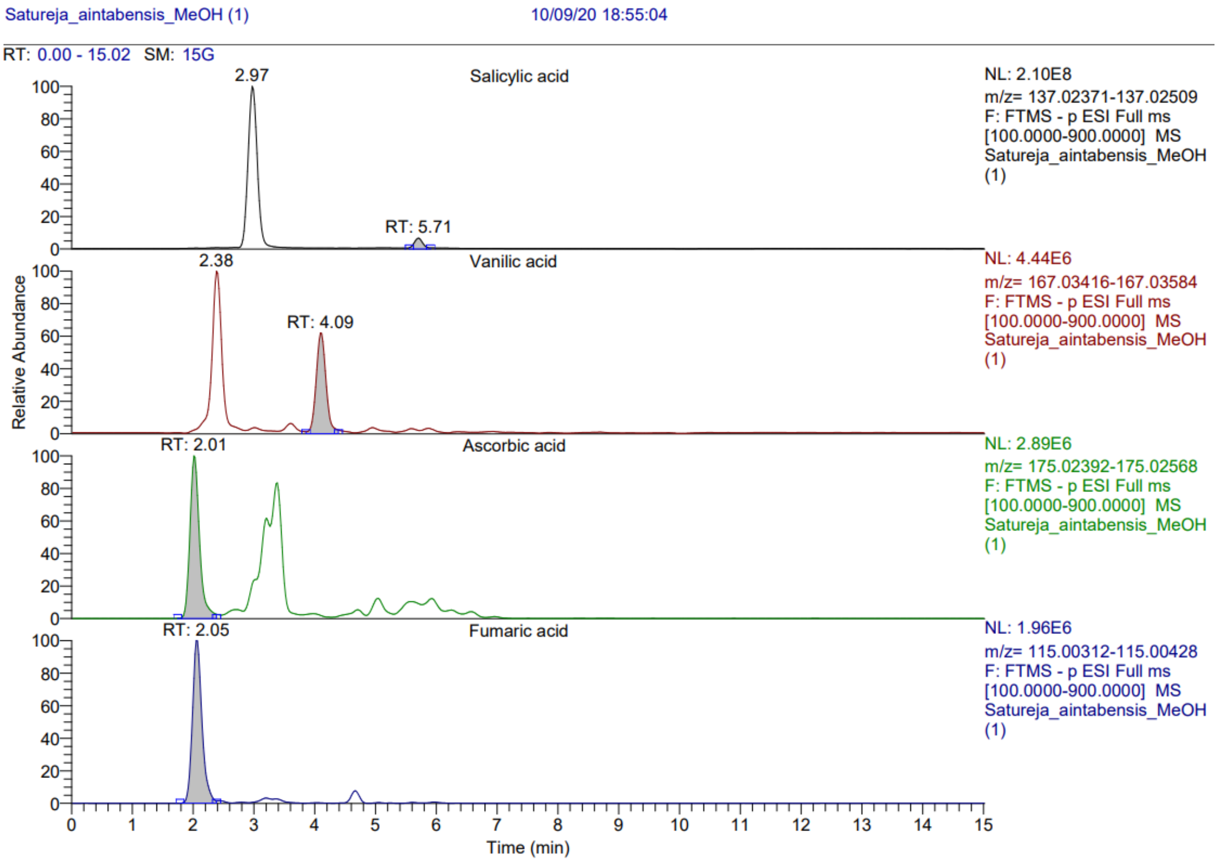

Figure S1. Continued

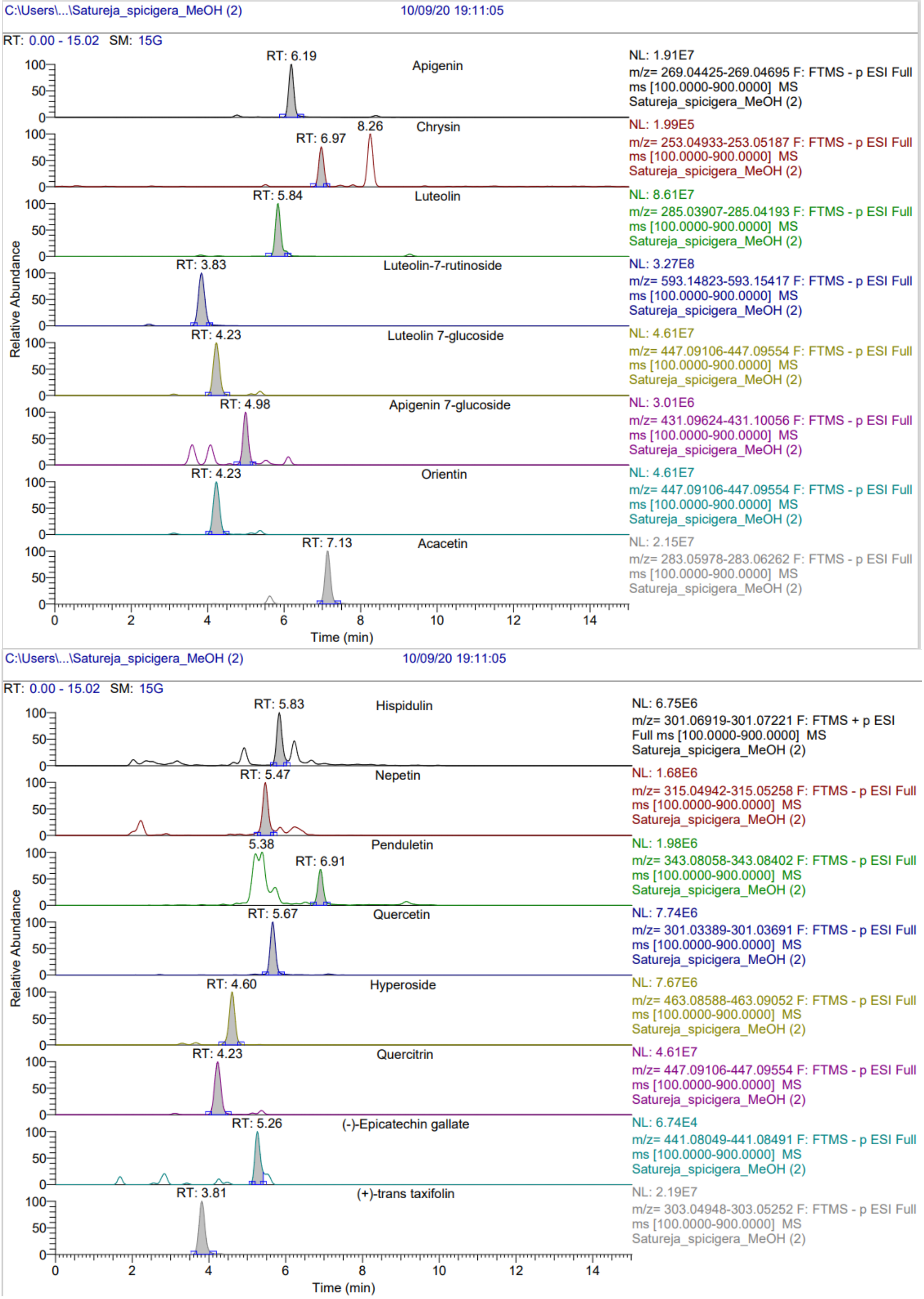

Figure S2. LC-HRMS chromatogram of *Satureja spicigera* (MeOH) extract

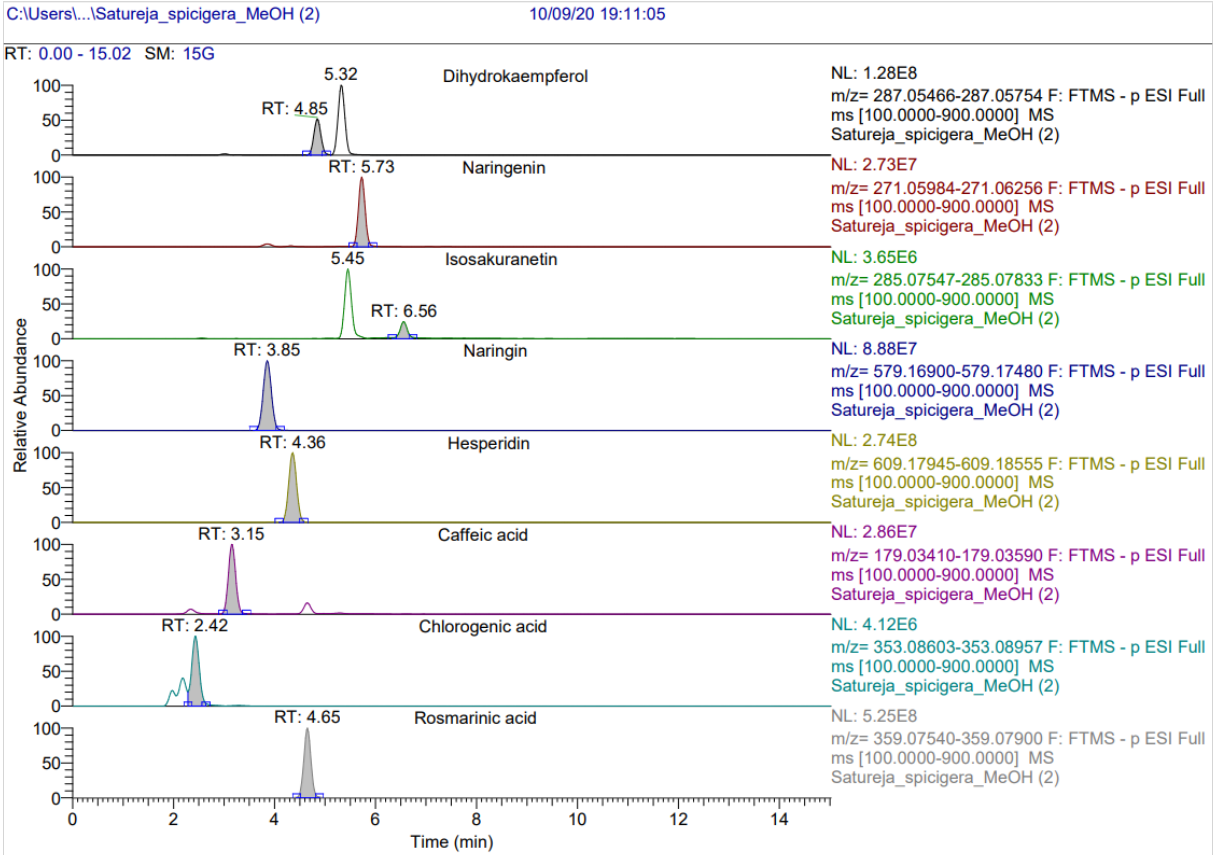

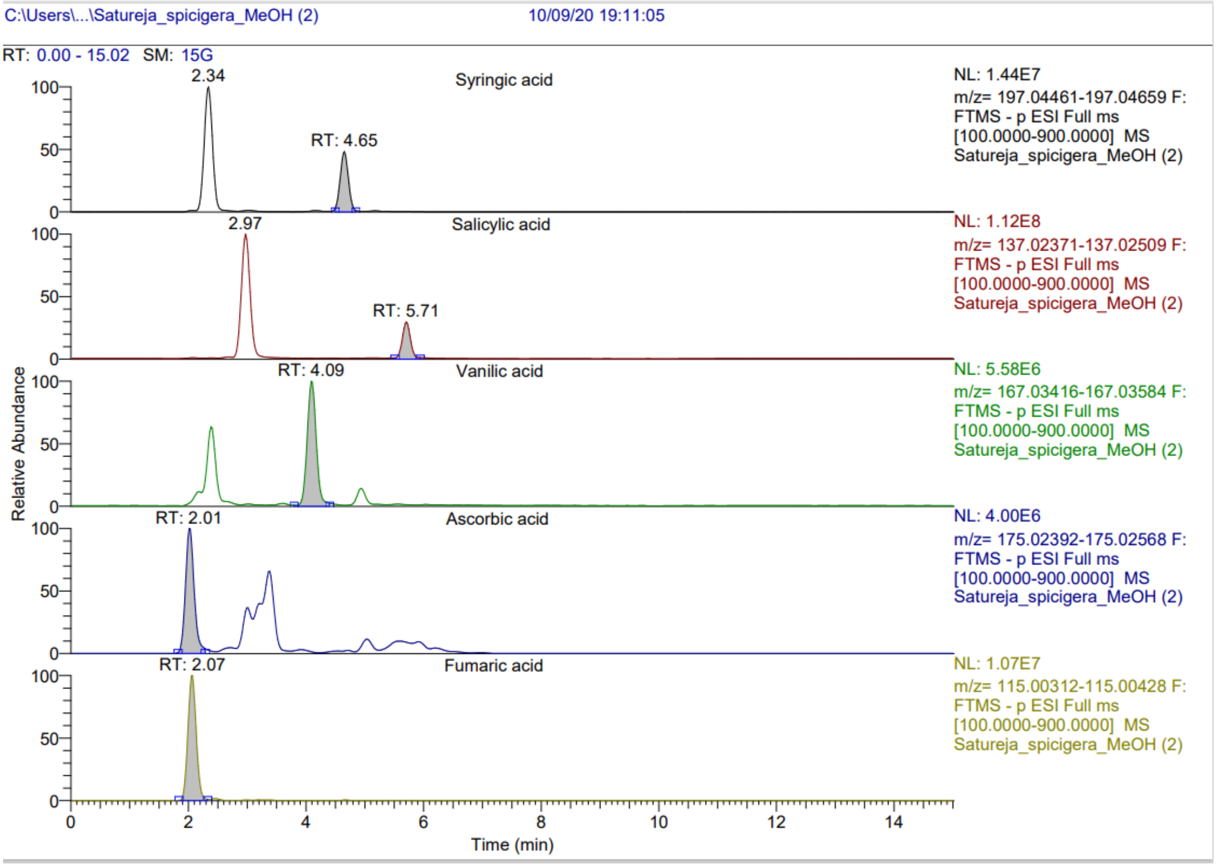

Figure S2. Continued

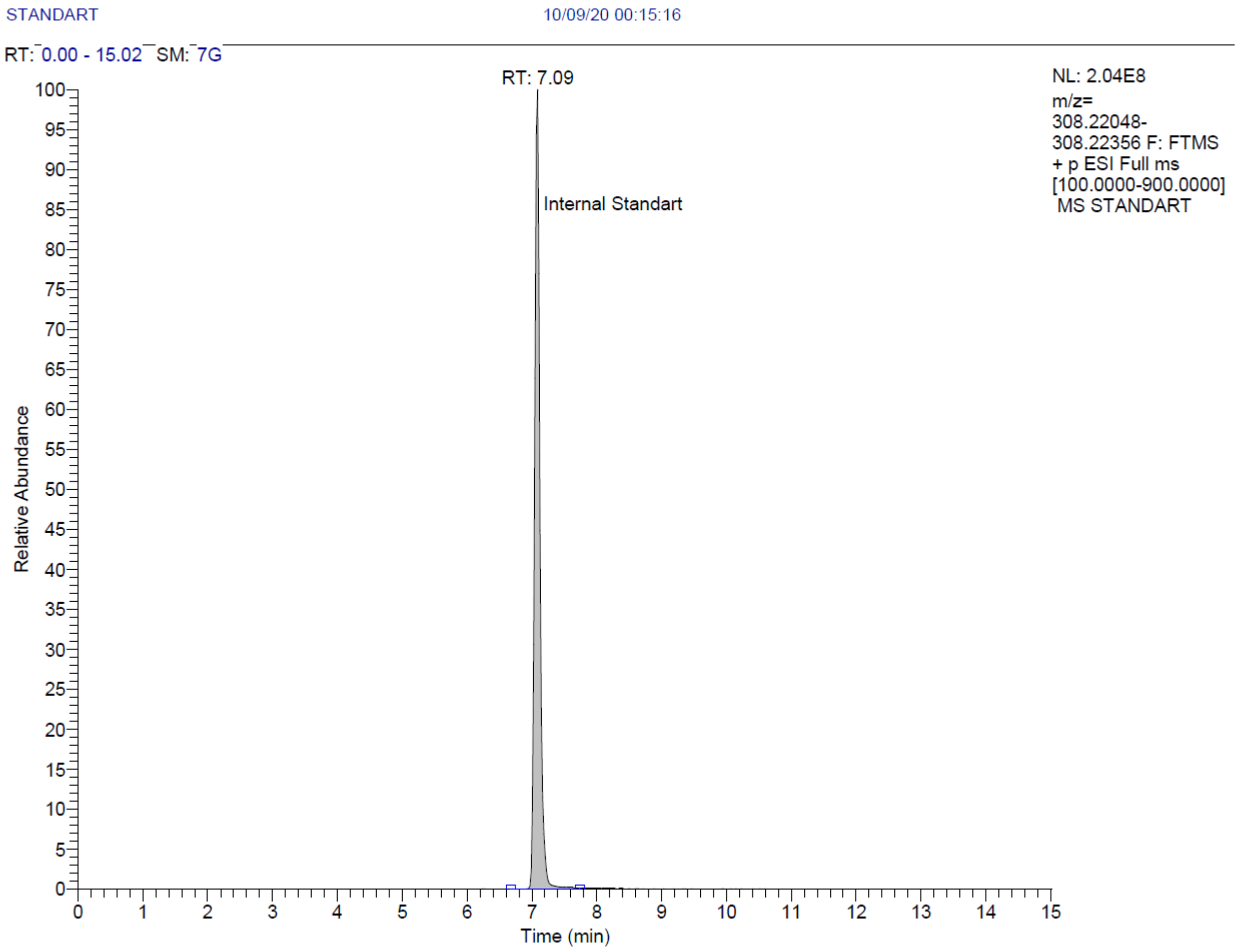

Figure S3. LC-HRMS chromatogram of internal standard (dihydrocapsaicin**)**
